# Philosophical Transactions of the Royal Society of London

**Published:** 1781-09

**Authors:** 


V. *Philofophical Tranfactions of the Royal Society of London.* Vol. 71, *for the year* 1781. Part 1. 4to. London, 1781, 226 pages, with 10 copper plates.

OF the fifteen papers contained in this work we fhall content ourfelves with particularly noticing the three following,

I. *Ex-*

I. *Experiments and obſervations on the ſpecific gravities and attractive powers of various ſaline ſubſtances. By* Richard Kirwan, *Eſq*; F. R. S.— The doctrine of chemical affinities hath of late been conſiderably improved by the labours of Bergman and Wenzel, but the order of theſe attractions has been the only point attended to by theſe philoſophers, as well as by the generality of preceding chemiſts. M. Morveau of Dijon is the only writer who has thought of aſcertaining the various degrees of force of chemical attraction, by which one body acts on various other bodies, or even on the ſame body in various circumſtances. He has, however, ſo ably ſhewn the advantages ariſing from ſuch an inquiry, that the author of the paper before us having been induced to make it the ſubject of his attention, has been enabled to determine pretty exactly the proportion of the ingredients of many neutral ſalts, and the ſpecific gravity of the mineral acids in their pureſt ſtate and free from all water. The reſults of his experiments on this ſubject are given in ſeveral tables which the limits of our Journal will not allow us to inſert.—The lovers of philoſophical chemiſtry will receive much information from Mr. Kirwan's ingenious reſearches.

II. *An*

II. *An account of the violent ſtorm of lightning at Eaſt-bourn in Suſſex, Sept.* 17, 1780. *Communicated by* Owen Saluſbury Brereton, *Eſq*; F. R. and A. S.

The melancholy accident here deſcribed happened in a houſe rented by James Adair, Eſq; near the ſea. The morning was very ſtormy, and at nine o'clock a black cloud appeared, out of which Mr. Adair ſaw ſeveral balls of fire drop into the ſea ſucceſſively, as he was approaching the window in a one-pair of ſtairs room; and very ſoon after, as he was ſtanding at it with his hands claſped, a flaſh of fire forced them aſunder, and threw him ſeveral yards upon the floor, where he remained motionleſs for a conſiderable time. The right ſleeve of his coat, waiſtcoat, and ſhirt, and likewiſe the right ſide of his breeches, were torn intirely open, as if by a dog, and his right arm, right ſide and thigh were miſerably ſcorched, and the fleſh torn. His right ſhoe and ſtocking likewiſe were torn, and one of his toes ſplit almoſt to the bone. Under the room Mr. Adair was in, on the parlour floor, were his coachman, butler, and footman. The coachman was going to open a glaſs door to go towards the ſea, and was ſtruck dead. His body was totally black. His cloaths and the caul of his wig, and cravat, cravat, were much torn; but no wound was to be ſeen in any part of his body.—The footman was dreſſing his hair near a window, when he was thrown dead on the ground. He appeared much ſcorched, bruiſed, and black. He had a very large wound in his ſide, which penetrated near his heart; but very little, if any, blood came from it. It is remarkable, that although the bodies of theſe two perſons lay unburied from Sunday till Tueſday, all their limbs remained perfectly flexible. The butler was a yard or two behind the coachman, and going out with a teleſcope in his hand, which was forced in pieces from him, his hat and wig were thrown to ſome diſtance, and he perceived a violent preſſure on his ſkull and on his back, but was no otherwiſe hurt. He had a ſilver watch with a ſilver chain, which received no damage. It was obſerved likewiſe, that a ſilver buckle in Mr. Adair's ſhoe was not in the leaſt hurt, whereas his watch chain, which was of ſteel, the braſs button of his breeches, and a penknife and key in his right ſide breeches pocket, had ſeveral marks of fuſion upon them; and the enamelled face of a ſilver watch in the coachman's fob was broken to pieces, and the links of his ſteel chain faſtened together.

In the room over Mr. Adair's a young lady was dreffing and her maid attending. They were both driven to a diftant part of the room, and rendered infenfible for fome time, but not hurt.

III. *An account of the Harmattan, a fingular African wind. By* Matthew Dobfon, M. D. F. R. S. *Communicated by* John Fothergill, M. D. F. R. S.

The Harmattan is a periodical wind which blows from the interior parts of Africa towards the Atlantic ocean, and poffeffes fuch extraordinary properties, as to merit the attention of the naturalift, making a curious and important article in the hiftory and theory of the winds.

The account here given of it is collected from obfervations communicated to the author by Mr. Norris, an intelligent gentleman, who has frequently vifited the coaft of Africa.

This fingular wind comes on indifcriminately at any hour of the day, or at any period of the moon, and continues fometimes only a day or two, fometimes fifteen or fixteen days. There are generally three or four returns of it every feafon. The peculiarities that attend it are a fog or haze, extreme drynefs, and falubrity. The fog is fo great as to make even near objects obfcure, and the fun concealed the greateft part of the the day, appears only during a few hours about noon, and then of a mild red, exciting no painful fenfation on the eye. No dew falls during the continuance of this wind. Vegetables of every kind are much injured and even deftroyed by it if it continues long. Such is the extreme drynefs produced by it that covers of books, fhut up in trunks among cloaths, are bent as if they had been expofed to the fire. Houfhold furniture is alfo much damaged by it; the pannels of doors fplit, and the joints of a well-laid floor of feafoned wood open fufficiently to lay one's finger in them; but become as clofe as before on the ceafing of the Harmattan.

The parching effects of this wind are likewife evident on the external parts of the body. The eyes, noftrils, lips, and palate are rendered dry and uneafy, and drink is often required, not fo much to quench thirft, as to remove a painful aridity in the fauces. The lips and nofe become fore and even chopped, and if the Harmattan continues four or five days, the fcarf fkin peels off, firft from the hands and face and afterwards from the reft of the body, if it continues a day or two longer.—Salt of tartar, moiftened *ad deliquium*, became perfectly dry in two or three hours when expofed to the Harmattan. Such is the salubrity of this wind that it stops the progress of epidemical and contagious diseases, and patients labouring under fevers or fluxes generally recover during its continuance. It is likewise noted for contributing much to the cure of ulcers, as well as cutaneous eruptions. This differs from the account given by Dr. Lind, who, speaking of the fatal and malignant effects of this wind, observes, "that its noxious vapours "are destructive to blacks as well as whites, "and that the mortality which it occasions "is in proportion to the density and duration of the fog." Our author, who is aware of this contradiction, supposes that there may be instances in which the Harmattan comes loaded with the effluvia of a putrid marsh, and that in such the nature of the wind may be so changed as to become noxious.

According to Dr. Lind the Harmattan arises from the conflux of several rivers about Benin; but Mr. Norris conjectures, that it takes its source from a part of Africa about the 15th deg. of north lat. and the 25th deg. of east long.—Our author thinks it probable, that the disagreeable Levant wind of the Mediterranean proceeds from the same part of the continent of Africa.—The paper concludes with an account of the manmanner in which the Fantees, a nation on the Gold coaſt, divide their year.

The papers that form the remainder of the contents are, *A natural hiſtory and deſcription of the Tyger Cat of the Cape of Good Hope, by* J. R. Forſter, L. L. D. F. R. S. and A. S.——*Eſſay on a new method of applying the ſcrew, by Mr.* William Hunter, *ſurgeon.*——*An account of the Turkey, by* Thomas Pennant, *Eſq*; F. R. S.——*Account of a Nebula in Comâ Berenices, by* Edw. Pigott, *Eſq*;——*Double ſtars diſcovered in* 1779, *at Frampton-houſe, Glamorganſhire, by* N. Pigott, *Eſq*; F. R. S.——*An account of the Ganges and Burrampooter rivers, by* James Rennell, *Eſq*; F. R. S.——*Obſervations on the rotation of the planets round their axes, by Mr.* W. Herſchel.——*Some account of the Termites, which are found in Africa and other hot climates, by Mr.* H. Smeathman.——*An account of ſeveral Earthquakes felt in Wales, by* Tho. Pennant, *Eſq*; F. R. S.——*Extract of a letter from the right hon. Earl* Stanhope, F. R. S. containing obſervations concerning the roots of adfected equations.——*Extract of two meteorological journals of the weather obſerved on the coaſt of Labradore, by Mr.* De la Trobe.——*Meteorological journal for* 1780, *kept at the houſe of the Royal Society.*——Prefixed to the volume are a liſt of the Society, and a

ſpeech speech delivered by the president at their last anniversary.

